# Early Addition of Evolocumab to Statin Treatment in Patients with Acute Coronary Syndrome and Multivessel Disease Undergoing Percutaneous Coronary Intervention

**DOI:** 10.31083/j.rcm2409270

**Published:** 2023-09-25

**Authors:** Yahao Zhang, Anjian Zhang, Yong Wu, Yanghui Zhang, Weiwei Hu, Penglei Chen, Kui Chen, Jiandong Ding

**Affiliations:** ^1^Department of Cardiology, Zhongda Hospital, Southeast University, 210009 Nanjing, Jiangsu, China; ^2^Department of Cardiology, The First Affiliated Hospital of Zhengzhou University, 450052 Zhengzhou, Henan, China

**Keywords:** lipid lowering, acute coronary syndrome, multivessel disease, PCSK9 inhibitor, percutaneous coronary intervention

## Abstract

**Background:**

Evolocumab has been demonstrated to significantly reduce ischemic cardiovascular events in patients with stable coronary heart disease. However, it is currently unclear whether this benefit extends to patients with acute coronary syndrome (ACS) and multivessel disease (MVD) undergoing percutaneous coronary intervention (PCI). The objective of this study was to assess the safety, efficacy and feasibility of the early addition of evolocumab to statin treatment for ACS patients with MVD undergoing PCI.

**Methods:**

The authors conducted a multicenter, retrospective cohort study involving 1199 ACS patients with MVD undergoing PCI and with elevated low-density lipoprotein cholesterol (LDL-C) levels. Patients were divided into an evolocumab group or a standard-of-care group based on evolocumab use or not. The 18-month primary efficacy endpoint was a composite of ischemic stroke, death from cardiac causes, recurrent myocardial infarction (MI), unplanned coronary revascularization or unstable angina requiring hospitalization. The principal secondary efficacy endpoint was a composite of ischemic stroke, death from cardiac causes or recurrent MI.

**Results:**

After propensity score matching, the addition of evolocumab to statin treatment lowered LDL-C levels by 42.62% compared with statin therapy alone at 18 months, from a mean baseline level of 3.37–0.75 mmol/L (*p* < 0.001). Relative to standard therapy, evolocumab added to statins was associated with significant reductions in the primary efficacy endpoint (8.3% *vs*. 13.3%; adjusted hazard ratio [HR], 0.60; 95% confidence interval [CI], 0.39 to 0.91; *p* = 0.017) and the principal secondary efficacy endpoint (6.1% *vs*. 10.2%; adjusted HR, 0.61; 95% CI, 0.37 to 0.99; *p* = 0.048) after multivariable Cox regression adjustment. The treatment effect of evolocumab was consistent across all prespecified subgroups. There were no significant between-group differences in terms of adverse events.

**Conclusions:**

In ACS patients with MVD taken for PCI, early initiation of evolocumab along with statin treatment was associated with a significant reduction in LDL-C levels and a reduced risk of recurrent cardiovascular events.

**Clinical Trial Registration:**

Chinese Clinical Trials Registry, identifier ChiCTR2000035165. Date: 2 August 2020. URL: https://www.chictr.org.cn/.

## 1. Introduction

In spite of the availability of many evidence-based therapies, patients presenting with acute coronary syndrome (ACS) undergoing percutaneous coronary intervention (PCI) remain at increased risk of recurrent ischemic cardiovascular events, especially in the acute phase following the index event [1, 2, 3]. The excessive risk is more pronounced in patients with multivessel disease (MVD). Multiple large-scale clinical trials have demonstrated that patients with MVD are at significantly elevated risk of myocardial infarction (MI), major adverse cardiovascular events and all-cause mortality [4, 5, 6, 7].

Low-density lipoprotein cholesterol (LDL-C) is an accepted and independent risk factor for cardiovascular disease. The 2019 European Society of Cardiology (ESC) and European Atherosclerosis Society (EAS) guideline identified patients with recent ACS as extremely high risk and recommended an LDL-C target of <1.4 mmol/L, in whom a high-intensity statin is recommended to be initiated in the acute phase following the index event [8]. In clinical practice, many ACS patients fail to achieve the guideline-recommended LDL-C target regardless of potent and stable statin treatment [9, 10]. In addition, the therapeutic benefits of statins are limited by the delayed onset of action, statin intolerance [11], the residual high risk of recurrent cardiovascular events [12], as well as inertia with regard to dose maximization [13]. This cumulative data emphasize the potential necessity for developing alternative intensive lipid-lowering treatments to further reduce the risk of recurrence of cardiovascular events.

Proprotein convertase subtilisin-kexin type 9 (PCSK9) inhibitors, as new lipid-lowering drugs, can rapidly and substantially reduce LDL-C levels. Evolocumab has been demonstrated to significantly lower major cardiovascular events in subjects with stable atherosclerotic cardiovascular disease (ASCVD) in the secondary prevention setting [14, 15, 16]. Nevertheless, the safety, efficacy and feasibility for the early addition of the PCSK9 inhibitor evolocumab to statin treatment in ACS patients with MVD undergoing PCI are presently unclear. In the current study, we tested the hypothesis that evolocumab combined with statins would result in a more favorable reduction in recurrent cardiovascular events as compared to statins alone among patients presenting with ACS within days and with MVD undergoing PCI.

## 2. Methods

### 2.1 Study Design and Patients

In this multicenter, retrospective cohort study, we screened consecutive ACS patients with MVD who underwent PCI at the First Affiliated Hospital of Zhengzhou University and Zhongda Hospital Southeast University from April 2019 to June 2020. Ethics committee approvals for this trial were obtained from all relevant centers, and the ethics committees waived the need for written informed consent.

Inclusion criteria: (1) patients hospitalized for a recent ACS with symptom onset <72 h; (2) patients with MVD (≥50% stenosis in ≥2 major epicardial coronary vessels) undergoing PCI; (3) patients with elevated LDL-C levels at presentation defined as one of the following: serum LDL-C ≥1.8 mmol/L on regular therapy with high-intensity statins for ≥4 weeks before admission, LDL-C ≥2.3 mmol/L on low-to-moderate-intensity statins for ≥4 weeks prior to admission, or LDL-C ≥3.2 mmol/L without regular statin treatment; (4) patients aged between 40 and 85 years. The intensity of statins was categorized as low, moderate or high intensity according to the 2018 American Heart Association (AHA)/American College of Cardiology (ACC) Guideline on the Management of Blood Cholesterol [17]. The determination of LDL-C thresholds was based on the criteria of the EVOPACS (EVOlocumab for Early Reduction of LDL-cholesterol Levels in Patients With Acute Coronary Syndromes) trial [18].

Exclusion Criteria: (1) New York Heart Association class III or IV; (2) uncontrolled ventricular tachycardia; (3) severe renal or hepatic dysfunction; (4) malignancy within the last 5 years; (5) statin intolerance; (6) prior use of PCSK9 inhibitors; (7) stable coronary heart disease; (8) presence of severe non-cardiovascular disease.

### 2.2 Study Interventions

Patients were divided into an evolocumab group or a standard-of-care group based on evolocumab use or not. Evolocumab was administered for 18 months at a dose of 140 mg every 2 weeks via subcutaneous injection.

Patients in the two groups received maximally tolerated statin treatment (rosuvastatin ≥10 mg or atorvastatin ≥20 mg per day) after admission. If the LDL-C target was not reached after 4-6 weeks of statin therapy, a high-intensity statin or ezetimibe will be recommended by the treating physician. The use of other cardiovascular medications was permitted in accordance with professional guidelines. Decisions concerning the arterial access site, use of intra-aortic balloon pump, revascularization strategy and stent type were at the discretion of the attending interventional cardiologist. Blood sampling was performed the morning after admission to assess LDL-C levels at baseline. Patients underwent visits at months 1, 6, 12 and 18 during the study period.

### 2.3 Clinical Endpoints

The 18-month primary efficacy endpoint was the composite of recurrent MI, ischemic stroke, death from cardiac causes, unplanned coronary revascularization or unstable angina requiring hospitalization. The principal secondary efficacy endpoint was the composite of recurrent MI, ischemic stroke or death from cardiac causes at 18 months. Other secondary endpoints included the components of the primary efficacy endpoint, all-cause death, target vessel MI as well as non-target vessel MI. The safety endpoints included laboratory abnormalities, muscle-related events, neurocognitive disorders, cataracts and new-onset diabetes. The consistency of the evolocumab treatment effect for the primary efficacy endpoint, compared with the standard treatment, was examined in nine pre-specified subgroups. Follow-up data for clinical endpoints were obtained through a review of hospital records, telephone calls, or both.

### 2.4 Sample Size and Statistical Analysis

Our initial hypothesis was that at 18 months, patients who received evolocumab would experience a lower rate of primary endpoint events compared to those who received standard-of-care treatment. We estimated that the number of subjects in the control group would be three times that of the evolocumab group. Assuming a rate of primary endpoint events of 14.0% at 18 months in the standard-of-care group, an overall sample size of 1192 subjects (of which 882 are in the control group and 310 are in the evolocumab group) would provide 80% power at a 0.05 significance level to detect a hazard ratio of 0.63.

All analyses were performed in accordance with the intention-to-treat principle. Continuous data were presented as mean with standard deviation (SD) and were tested using the Student’s *t*-test (or the Mann–Whitney test for non-normal data). Categorical data were presented as frequencies and percentages and were tested using the χ^2^ test (or the Fisher exact test for sparse data). Time-to-event data were presented with the use of Kaplan–Meier estimates. Hazard ratios (HRs) and 95% confidence intervals (CIs) were calculated with the use of a multivariable Cox regression model. Covariates included age, sex, index ACS, weight, cardiac arrest, diabetes mellitus, current smoking, previous PCI, hypertension, previous coronary artery bypass grafting, previous MI, prior stroke, prior thrombolytic therapy, peripheral vascular disease, chronic obstructive pulmonary disease, family history of coronary heart disease (CHD), estimated glomerular filtration rate, arterial access site, revascularization strategy, intra-aortic balloon pump, number of diseased vessels, thrombolysis in myocardial infarction (TIMI) flow 0 to 1 before PCI, treated vessels, stent number per patient, thrombus in the treated lesion, overall stent length per patient, type of anticoagulant used during PCI, thrombus aspiration, and full procedural success. Pre-specified subgroup analyses were conducted using a Cox proportional hazards model, with factors including subgroups, treatment groups, and interaction between treatment groups and subgroups.

To minimalize selection bias and potential confounding between the 2 treatment groups, we conducted rigorous adjustments on the baseline and procedural characteristics using propensity score matching in a 1:1 ratio without replacement. Statistical analyses were done with the use of STATA version 16.0 (Stata Corp., College Station, TX, USA) and SPSS version 25.0 (IBM Corp., Armonk, NY, USA), and a 2-sided *p* value of <0.05 was required for statistical significance.

## 3. Results

### 3.1 Patients

The baseline characteristics of the two groups were generally well balanced except for the increased incidence of a family history of CHD in the evolocumab group (26.5% *vs*. 20.2%, *p* = 0.020). The mean age was 62.1 ± 10.1 years, 59.5% of patients were men, 20.8% had a previous MI and 66.0% had not received stable statin treatment within 4 weeks prior to admission (Table 1).

**Table 1.** Baseline characteristics*.

Characteristic		All patients				Propensity-matched patients			
		Evolocumab (N = 313)	Control (N = 886)	χ^2^/t/F	*p* value	Evolocumab (N = 313)	Control (N = 313)	χ^2^/t/F	*p* value
Age, yr		61.9 ± 10.6	62.1 ± 9.9	0.332	0.740	61.9 ± 10.6	61.9 ± 10.0	0.054	0.957
Weight, kg		73.0 ± 11.6	74.2 ± 12.1	1.507	0.132	73.0 ± 11.6	72.1 ± 10.8	0.999	0.318
Men, No. (%)		196 (62.6)	518 (58.5)	1.658	0.198	196 (62.6)	183 (58.5)	1.130	0.288
Clinical presentation, No. (%)				1.404	0.496			2.958	0.085
	NSTEMI	93 (29.7)	269 (30.4)			93 (29.7)	84 (26.8)		
	STEMI	76 (24.3)	187 (21.1)			76 (24.3)	57 (18.2)		
	Unstable angina	144 (46.0)	430 (48.5)			144 (46.0)	172 (55.0)		
Cardiac arrest, No. (%)		12 (3.8)	30 (3.4)	0.137	0.711	12 (3.8)	5 (1.6)	2.177	0.140
Current smoker, No. (%)		95 (30.4)	285 (32.2)	0.352	0.553	95 (30.4)	89 (28.4)	0.277	0.599
Diabetes, No. (%)		88 (28.1)	293 (33.1)	2.619	0.106	88 (28.1)	89 (28.4)	0.008	0.929
	Insulin-dependent	31 (9.9)	99 (11.2)	0.386	0.535	31 (9.9)	32 (10.2)	0.018	0.894
Hypertension, No. (%)		204 (65.2)	575 (64.9)	0.008	0.930	204 (65.2)	212 (67.7)	0.459	0.498
Previous stroke, No. (%)		17 (5.4)	75 (8.5)	3.005	0.083	17 (5.4)	27 (8.6)	2.445	0.118
Previous coronary artery bypass grafting, No. (%)		10 (3.2)	33 (3.7)	0.188	0.665	10 (3.2)	9 (2.9)	0.054	0.816
Prior myocardial infarction, No. (%)		64 (20.4)	185 (20.9)	0.026	0.871	64 (20.4)	79 (25.2)	2.039	0.153
Previous percutaneous coronary intervention, No. (%)		61 (19.5)	166 (18.7)	0.085	0.770	61 (19.5)	68 (21.7)	0.478	0.489
Peripheral vascular disease, No. (%)		17 (5.4)	36 (4.1)	1.025	0.311	17 (5.4)	15 (4.8)	0.132	0.717
Family history of coronary heart disease, No. (%)		83 (26.5)	179 (20.2)	5.400	0.020	83 (26.5)	72 (23.0)	1.038	0.308
Chronic obstructive pulmonary disease, No. (%)		18 (5.8)	58 (6.5)	0.247	0.620	18 (5.8)	19 (6.1)	0.029	0.865
Estimated glomerular filtration rate, mL/min		84.6 ± 18.4	83.8 ± 23.5	0.607	0.544	84.6 ± 18.4	86.8 ± 22.5	1.355	0.176
Statin therapy before admission, No. (%)				3.877	0.144			1.165	0.558
	Low- or moderate-intensity	104 (33.2)	270 (30.5)			104 (33.2)	113 (36.1)		
	High-intensity	13 (4.2)	21 (2.4)			13 (4.2)	9 (2.9)		
	No statin	196 (62.6)	595 (67.2)			196 (62.6)	191 (61.0)		
Ezetimibe therapy, No. (%)		89 (28.4)	273 (30.8)	0.621	0.431	89 (28.4)	77 (24.6)	1.181	0.277
Prior thrombolytic treatment, No. (%)		8 (2.6)	23 (2.6)	0.001	0.969	8 (2.6)	4 (1.3)	1.359	0.244

* Data are mean ± SD or No. (%). NSTEMI, non-ST-segment elevation myocardial infarction; STEMI, ST-segment elevation myocardial infarction.

A total of 32 patients (10.2%) discontinued evolocumab treatment during follow-up (8.0% due to the high cost of evolocumab and 2.2% due to adverse events). At 18 months, complete follow-up data were available for 298 patients (95.2%) in the evolocumab group and for 836 patients (94.4%) in the standard-of-care group.

### 3.2 Procedural Characteristics

The information on the procedural characteristics is available in Table 2. Artery access was primarily radial in the two treatment groups, and the number of vessels treated was approximately the same in both groups. A total of 772 patients (64.4%) had triple-vessel disease, 340 patients (28.4%) had thrombus lesions, and 1172 patients (97.7%) underwent stent implantation. Full procedural success was similar in the two groups (95.8% in the evolocumab group *vs*. 97.9% in the control group) (Table 2).

**Table 2.** Procedural characteristics*.

Characteristic		All patients				Propensity-matched patients			
		Evolocumab (N = 313)	Control (N = 886)	χ ^2^_/t/F_	*p* value	Evolocumab (N = 313)	Control (N = 313)	χ ^2^_/t/F_	*p* value
Access, No. (%)				0.181	0.670			0.159	0.690
	Radial	280 (89.5)	800 (90.3)			280 (89.5)	283 (90.4)		
	Femoral	33 (10.5)	86 (9.7)			33 (10.5)	30 (9.6)		
Number of diseased vessels, No. (%)				0.005	0.942			0.172	0.678
	2-vessel disease	112 (35.8)	315 (35.6)			112 (35.8)	117 (37.4)		
	3-vessel disease	201 (64.2)	571 (64.4)			201 (64.2)	196 (62.6)		
Thrombus lesion, No. (%)		98 (31.3)	242 (27.3)	1.818	0.178	98 (31.3)	77 (24.6)	3.173	0.075
Treated vessel (s), No. (%)									
	Right coronary artery	124 (39.6)	311 (35.1)	2.040	0.153	124 (39.6)	113 (36.1)	0.822	0.365
	Left main	28 (8.9)	86 (9.7)	0.156	0.693	28 (8.9)	35 (11.2)	0.865	0.352
	Left circumflex	106 (33.9)	320 (36.1)	0.512	0.474	106 (33.9)	106 (33.9)	0.000	1.000
	Left anterior descending	181 (57.8)	545 (61.5)	1.315	0.252	181 (57.8)	201 (64.2)	2.686	0.101
Multi-vessel treatment, No. (%)		104 (33.2)	329 (37.1)	1.530	0.216	104 (33.2)	120 (38.3)	1.780	0.182
TIMI flow 0 to 1 prior to PCI, No. (%)		128 (40.9)	344 (38.8)	0.415	0.520	128 (40.9)	128 (40.9)	0.000	1.000
Intra-aortic balloon pump, No. (%)		14 (4.5)	31 (3.5)	0.607	0.436	14 (4.5)	13 (4.2)	0.039	0.844
Revascularization strategy, No. (%)				0.178	0.673			0.068	0.794
	Balloon angioplasty	8 (2.6)	19 (2.1)			8 (2.6)	7 (2.2)		
	Stent implantation	305 (97.4)	867 (97.9)			305 (97.4)	306 (97.8)		
Total stent length per patient, mm		47.3 ± 28.3	50.0 ± 29.9	1.383	0.167	47.3 ± 28.3	48.1 ± 29.2	0.341	0.734
Mean number of stents per patient		1.9 ± 0.9	2.0 ± 1.0	1.427	0.154	1.9 ± 0.9	2.0 ± 1.0	0.777	0.437
Anticoagulants during PCI				0.273	0.601			0.007	0.935
	Unfractionated heparin	188 (60.1)	547 (61.7)			188 (60.1)	189 (60.4)		
	Bivalirudin	125 (39.9)	339 (38.3)			125 (39.9)	124 (39.6)		
Full procedural success, No. (%)		300 (95.8)	867 (97.9)	3.593	0.058	300 (95.8)	303 (96.8)	0.406	0.524

* Data are mean ± SD or No. (%). PCI, percutaneous coronary intervention; TIMI, thrombolysis in myocardial infarction.

### 3.3 Propensity Score Matching Analyses

After propensity score matching was applied to the study population, 313 matched pairs of patients were identified for the comparison of evolocumab + statins versus statins alone. No significant differences were observed in baseline and procedure characteristics between the 2 groups, suggesting a substantial balance between evolocumab + statin and statin treatment (Tables 1 and 2).

### 3.4 Changes in Low-Density Lipoprotein Cholesterol Levels

**Fig. 1.**
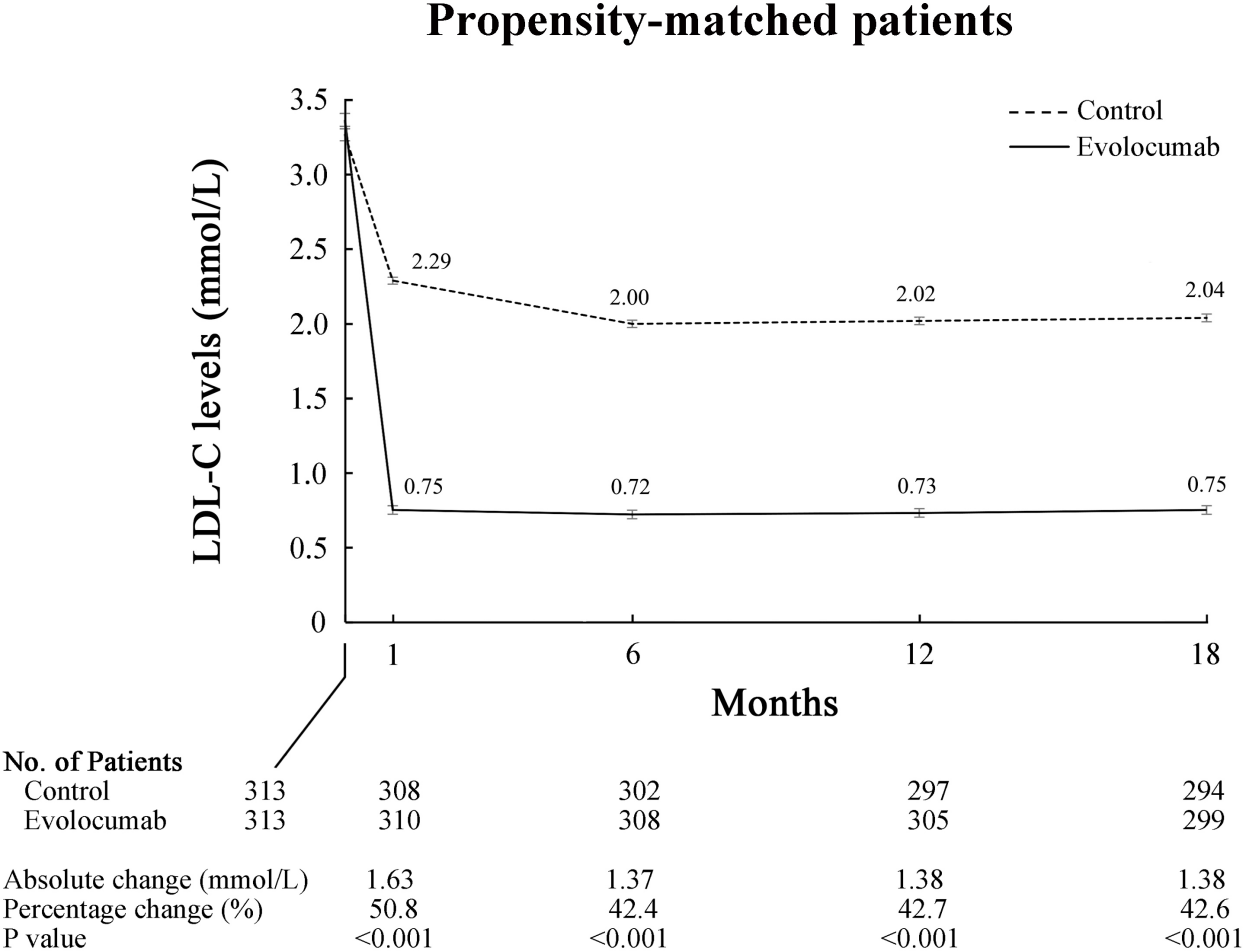
**Low-density lipoprotein cholesterol changes from baseline to 18 months in propensity-matched patients**. LDL-C, low-density lipoprotein cholesterol.

After propensity score matching, there was no statistically significant difference between groups in LDL-C levels at baseline (3.37 ± 0.72 *vs*. 3.27 ± 0.76 mmol/L, *p* = 0.106). The mean LDL-C percentage reduction from baseline to 18 months was –77.14% in the evolocumab group versus –34.52% in the standard-of-care group (*p* < 0.001) (Table 3). The reduction in LDL-C level was substantial at 1 month and this trend persisted at the 18-month follow-up (Fig. 1). At 18 months, LDL-C was reduced to less than 1.4 mmol/L in 271 patients (90.6%) in the evolocumab group, as compared to 24 patients (8.2%) in the standard-of-care group (Table 3).

**Table 3.** Changes in low-density lipoprotein cholesterol levels*.

Low-density lipoprotein cholesterol	All patients					Propensity-matched patients				
	Evolocumab (N = 313)	Control (N = 886)	Mean Difference (95% CI) ^a^	χ^2^/t	*p* value	Evolocumab (N = 313)	Control (N = 313)	Mean Difference (95% CI) ^a^	χ^2^/t	*p* value
At admission, mmol/L	3.37 ± 0.72	3.29 ± 0.79	–0.08 (–0.18 to 0.02)	1.65	0.098	3.37 ± 0.72	3.27 ± 0.76	–0.10 (–0.21 to 0.02)	1.62	0.106
Follow up at 18 months, mmol/L	0.75 ± 0.45	2.03 ± 0.51	1.28 (1.22 to 1.34)	41.07	<0.001	0.75 ± 0.45	2.04 ± 0.50	1.29 (1.21 to 1.36)	33.11	<0.001
Percent reduction from admission, %	–77.14 ± 14.18	–34.83 ± 22.31	42.30 (40.10 to 44.51)	37.61	<0.001	–77.14 ± 14.18	–34.52 ± 21.87	42.62 (39.64 to 45.60)	28.11	<0.001
Absolute reduction from admission, mmol/L	–2.63 ± 0.79	–1.26 ± 0.86	1.37 (1.27 to 1.48)	25.24	<0.001	–2.63 ± 0.79	–1.25 ± 0.89	1.38 (1.25 to 1.52)	19.99	<0.001
LDL-C <1.4 mmol/L at 18-month follow up, No. (%)	271 (90.6)	75 (8.9)	-	695.31	<0.001	271 (90.6)	24 (8.2)	-	403.32	<0.001

* Data are mean ± SD or No. (%). ^a^Control minus evolocumab. LDL-C, low-density lipoprotein cholesterol.

### 3.5 Other Lipid Measurements

**Fig. 2.**
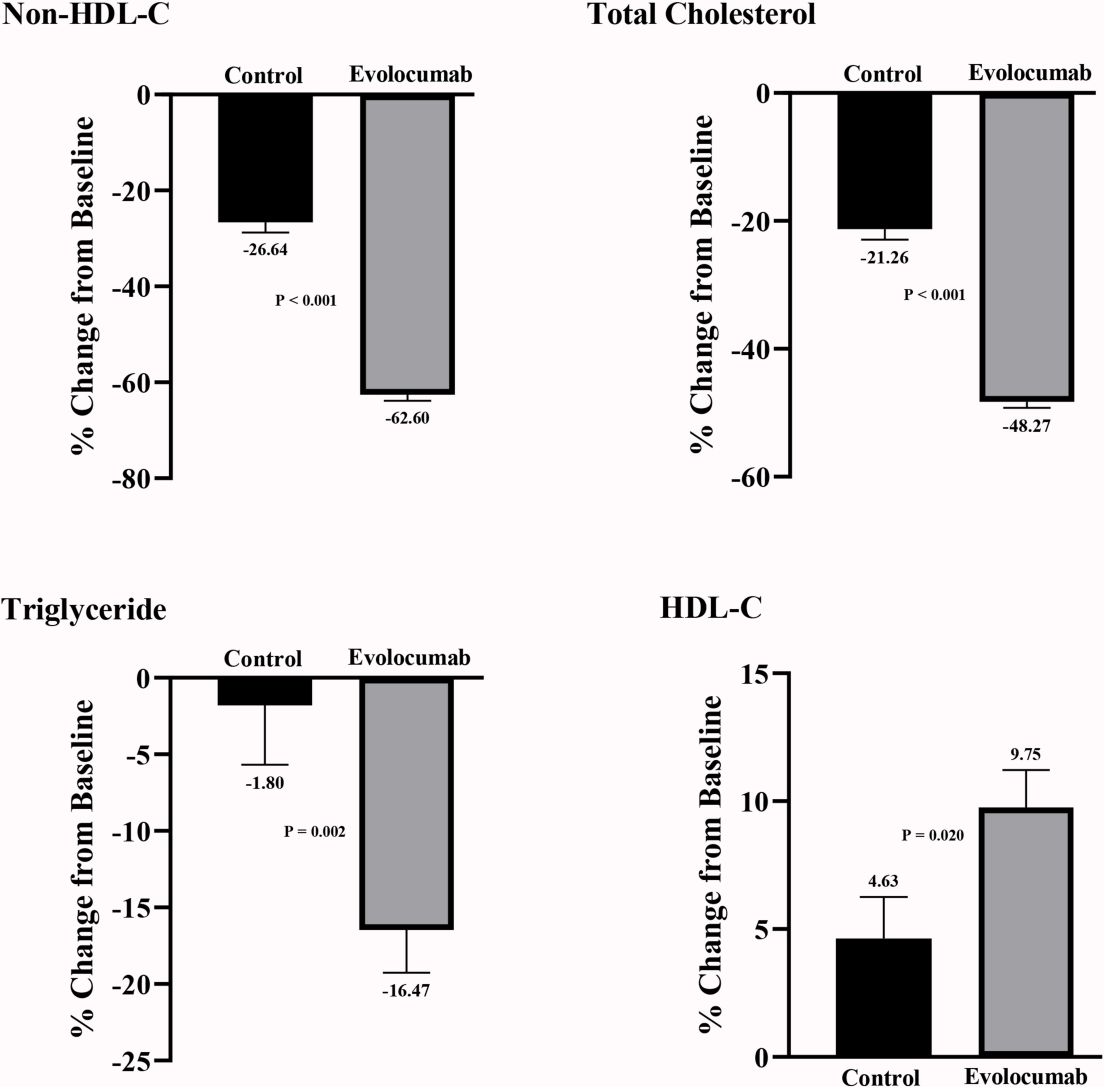
**Other lipid measurements in propensity-matched patients**. HDL-C, high-density lipoprotein cholesterol; Non-HDL-C, Non-high-density lipoprotein cholesterol.

Other lipid indicators are provided in Table 4 and Fig. 2. Evolocumab similarly reduced related parameters of atherogenic lipids. Compared with standard treatment, evolocumab had lowered total cholesterol levels by 27.01%, non-HDL-C levels by 35.96% and triglycerides levels by 14.67%. In contrast, evolocumab increased HDL-C levels by 5.12%.

**Table 4.** Changes in other lipids in propensity-matched patients*.

Lipid Measurements		Propensity-matched patients				
		Evolocumab (N = 313)	Control (N = 313)	Mean Difference (95% CI) ^a^	χ ^2^_/t_	*p* value
Total Cholesterol						
	At admission, mmol/L	5.38 ± 1.07	5.28 ± 1.17	–0.09 (–0.27 to 0.08)	1.04	0.300
	Follow up at 18 months, mmol/L	2.68 ± 0.73	3.97 ± 1.13	1.29 (1.14 to 1.44)	16.91	<0.001
	Percent reduction from admission, %	–48.27 ± 17.04	–21.26 ± 29.25	27.01 (23.26 to 30.77)	14.12	<0.001
	Absolute reduction from admission, mmol/L	–2.69 ± 1.29	–1.31 ± 1.68	1.38 (1.15 to 1.62)	11.54	<0.001
Non-HDL-C						
	At admission, mmol/L	4.28 ± 1.13	4.16 ± 1.17	–0.12 (–0.30 to 0.06)	1.29	0.199
	Follow up at 18 months, mmol/L	1.51 ± 0.76	2.83 ± 1.17	1.32 (1.17 to 1.47)	16.74	<0.001
	Percent reduction from admission, %	–62.60 ± 21.92	–26.64 ± 37.54	35.96 (31.13 to 40.78)	14.63	<0.001
	Absolute reduction from admission, mmol/L	–2.77 ± 1.30	–1.33 ± 1.70	1.44 (1.20 to 1.68)	11.87	<0.001
HDL-C						
	At admission, mmol/L	1.10 ± 0.31	1.12 ± 0.31	0.02 (–0.02 to 0.07)	1.00	0.317
	Follow up at 18 months, mmol/L	1.18 ± 0.30	1.14 ± 0.32	–0.03 (–0.08 to 0.02)	1.30	0.195
	Percent reduction from admission, %	9.75 ± 25.96	4.63 ± 28.89	–5.12 (–9.44 to –0.81)	2.33	0.020
	Absolute reduction from admission, mmol/L	0.08 ± 0.22	0.02 ± 0.28	–0.06 (–0.10 to –0.02)	2.85	0.005
Triglycerides						
	At admission, mmol/L	1.79 ± 0.72	1.75 ± 0.77	–0.03 (–0.15 to 0.08)	0.54	0.587
	Follow up at 18 months, mmol/L	1.32 ± 0.67	1.49 ± 0.87	0.17 (0.05 to 0.29)	2.70	0.007
	Percent reduction from admission, %	–16.47 ± 49.48	–1.80 ± 68.67	14.67 (5.27 to 24.06)	3.07	0.002
	Absolute reduction from admission, mmol/L	–0.46 ± 1.02	–0.26 ± 1.22	0.20 (0.02 to 0.38)	2.24	0.026

* Data are mean ± SD. ^a^Control minus evolocumab. HDL-C, high-density lipoprotein cholesterol.

### 3.6 Clinical Outcomes

**Fig. 3.**
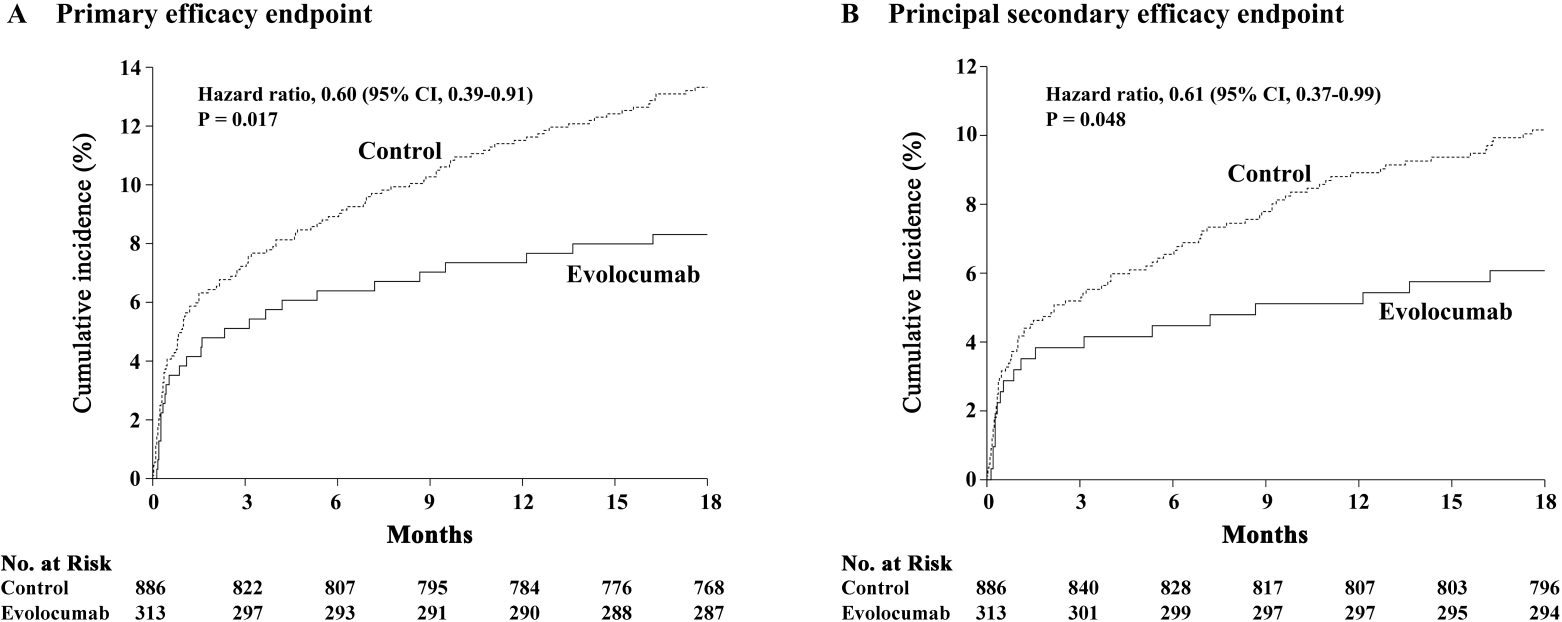
**Time-to-event curves for the primary efficacy endpoint (A) and the principal secondary efficacy endpoint (B) in patients with acute coronary syndrome and multivessel disease undergoing percutaneous coronary intervention**.

Before propensity score matching, relative to standard therapy, evolocumab added to statins was associated with a substantial reduction in the occurrence of the primary efficacy endpoint (8.3% *vs*. 13.3%; adjusted HR, 0.60; 95% CI, 0.39–0.91, *p* = 0.017) after multivariable Cox regression adjustment, predominantly driven by reductions in the rates of MI in both target and non-target vessels. Likewise, there was a significant reduction in the rate of the principal secondary efficacy endpoint (6.1% *vs*. 10.2%; adjusted HR, 0.61; 95% CI, 0.37–0.99, *p* = 0.048) (Fig. 3 and Table 5). In contrast, there were no significant differences between the treatment groups in terms of each component of the primary efficacy endpoint and all-cause death (Table 5).

**Table 5.** Primary and secondary outcomes*.

Outcome		All patients				Propensity-matched patients			
		Evolocumab (N = 313)	Control (N = 886)	Multivariable Adjusted Hazards Ratio (95% CI)	*p *value	Evolocumab (N = 313)	Control (N = 313)	Adjusted Hazards Ratio (95% CI)	*p* value
		No. (%)				No. (%)			
Primary efficacy endpoint: ischemic stroke, death from cardiac causes, recurrent MI, unplanned coronary revascularization or unstable angina requiring hospitalization		26 (8.3)	118 (13.3)	0.60 (0.39–0.91)	0.017	26 (8.3)	43 (13.7)	0.60 (0.37–0.98)	0.042
Principal secondary endpoint: ischemic stroke, death from cardiac causes or recurrent MI		19 (6.1)	90 (10.2)	0.61 (0.37–0.99)	0.048	19 (6.1)	33 (10.5)	0.56 (0.32–0.98)	0.044
Myocardial infarction		12 (3.9)	60 (6.9)	0.55 (0.30–1.02)	0.057	12 (3.9)	20 (6.5)	0.59 (0.29–1.20)	0.146
	Target vessel myocardial infarction	8 (2.6)	39 (4.5)	0.59 (0.28–1.26)	0.171	8 (2.6)	12 (3.9)	0.66 (0.27–1.61)	0.360
	Non-target vessel myocardial infarction	4 (1.3)	21 (2.4)	0.53 (0.18–1.55)	0.249	4 (1.3)	6 (1.9)	0.64 (0.18–2.26)	0.485
All-cause death		5 (1.6)	24 (2.7)	0.59 (0.22–1.54)	0.277	5 (1.6)	9 (2.9)	0.54 (0.18–1.61)	0.267
Death from cardiac causes		4 (1.3)	21 (2.4)	0.54 (0.18–1.56)	0.253	4 (1.3)	7 (2.2)	0.56 (0.17–1.92)	0.359
Ischemic stroke		4 (1.3)	15 (1.7)	0.79 (0.26–2.39)	0.679	4 (1.3)	7 (2.3)	0.57 (0.17–1.93)	0.363
Unplanned coronary revascularization		19 (6.1)	71 (8.1)	0.75 (0.45–1.24)	0.261	19 (6.1)	25 (8.1)	0.77 (0.42–1.40)	0.387
Unstable angina requiring hospitalization		3 (1.0)	12 (1.4)	0.74 (0.21–2.64)	0.648	3 (1.0)	4 (1.3)	0.75 (0.17–3.36)	0.709

* Percentages were calculated as estimates of cumulative incidence using the Kaplan-Meier method. MI, myocardial infarction.

After propensity score matching, results of clinical endpoints at 18 months were consistent with the primary adjusted analyses, which confirmed the beneficial effects of evolocumab + statins versus statins alone in terms of the primary and secondary efficacy endpoints (Table 5).

For the safety endpoints, no significant difference between the 2 groups was observed in the overall occurrence of adverse events. Similarly, the occurrences of laboratory abnormalities, muscle-related events, cataracts, neurocognitive disorders and new-onset diabetes did not differ substantially between the study groups (Table 6).

**Table 6.** Adverse events and laboratory results*.

Outcome		All patients				Propensity-matched patients			
		Evolocumab (N = 313)	Control (N = 886)	χ ^2^	*p* value	Evolocumab (N = 313)	Control (N = 313)	χ ^2^	*p* value
Adverse events, No. (%)									
	Muscle-related event	10 (3.2)	25 (2.8)	0.114	0.736	10 (3.2)	7 (2.2)	0.544	0.461
	Neurocognitive disorder	3 (1.0)	6 (0.7)	0.246	0.620	3 (1.0)	4 (1.3)	0.144	0.704
	New-onset diabetes	12 (3.8)	40 (4.5)	0.258	0.611	12 (3.8)	13 (4.2)	0.042	0.838
	Cataract	2 (0.6)	8 (0.9)	0.195	0.659	2 (0.6)	2 (0.6)	0.000	1.000
Laboratory results, No./total No. (%)									
	ALT >3 × ULN	4/306 (1.3)	14/857 (1.6)	0.158	0.691	4/306 (1.3)	7/303 (2.3)	0.864	0.353
	Creatine kinase >5 × ULN	2/305 (0.7)	4/852 (0.5)	0.151	0.698	2/305 (0.7)	2/301 (0.7)	0.000	0.989

* Data are No. (%) or No./total No. (%). ALT, alanine aminotransferase; ULN, upper limit of normal.

### 3.7 Subgroup Analyses

**Fig. 4.**
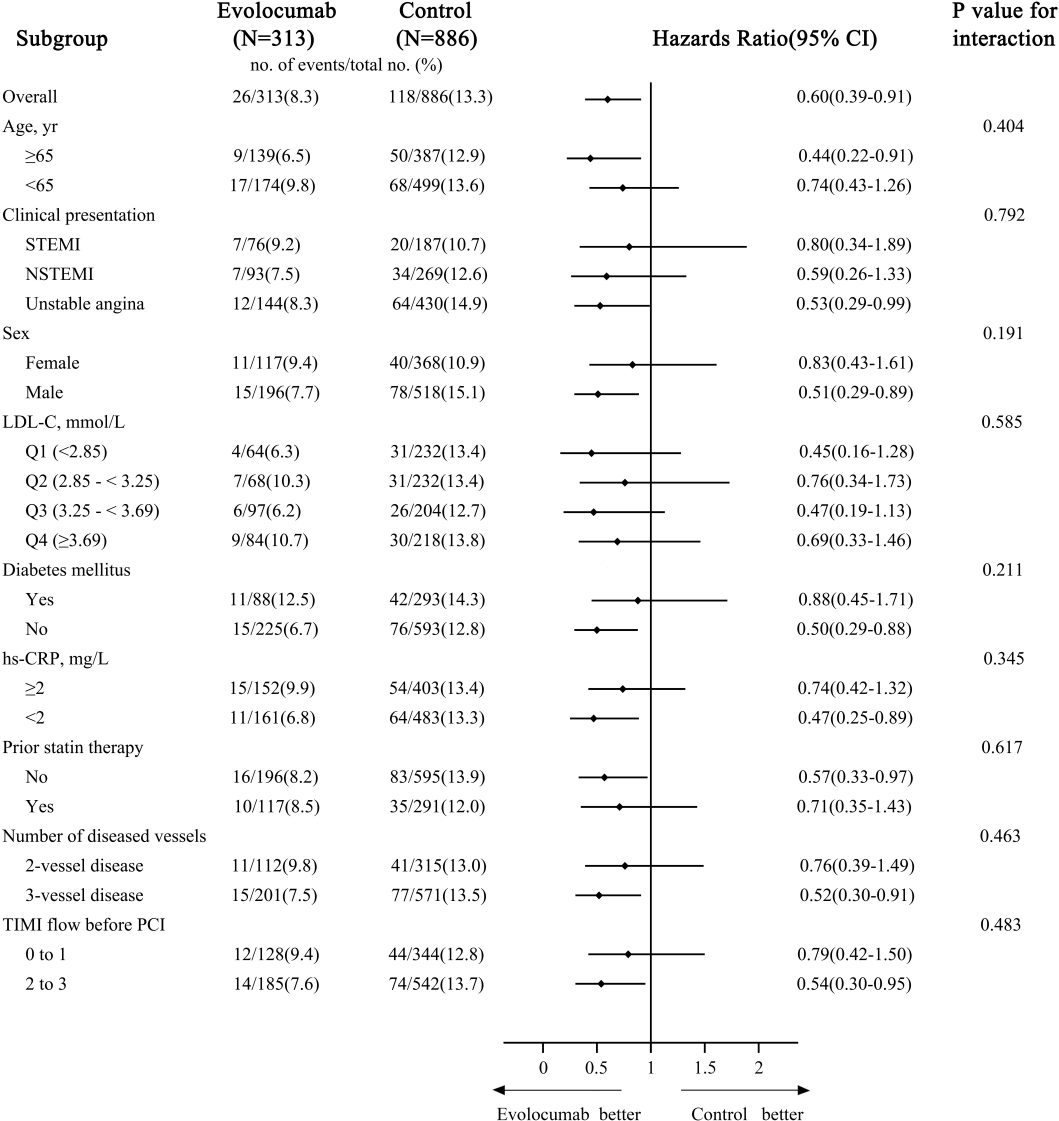
**Subgroup analyses for the primary efficacy endpoint at 18 months**. STEMI, ST-segment elevation myocardial infarction; NSTEMI, non-ST-segment elevation myocardial infarction; LDL-C, low-density lipoprotein cholesterol; hs-CRP, hypersensitive C-reactive protein; PCI, percutaneous coronary intervention; TIMI, thrombolysis in myocardial infarction.

The effect of evolocumab on the 18-month primary efficacy endpoint was consistent across 9 pre-specified subgroups, including risk populations defined based on age (≥65 years *vs*. <65 years) and based on the absence or presence of diabetes mellitus (Fig. 4). For all prespecified subgroups, there were no significant interactions between any subgroup and treatment groups with respect to the primary efficacy endpoint.

## 4. Discussion

In the present clinical trial evaluating in-hospital use of evolocumab in ACS patients with MVD undergoing PCI, the addition of evolocumab at 140 mg every 2 weeks to statin treatment, compared with statins alone, resulted in sustained reductions in LDL-C levels throughout the follow-up period. At 18 months, the primary efficacy endpoint and principal secondary efficacy endpoint were substantially reduced by evolocumab plus statins compared with statin therapy alone. Regarding safety outcomes, there were no statistically significant differences between groups in the 18-month rates of adverse events.

Genetic and epidemiological data have identified a causal role for LDL-C in ASCVD [19, 20]. A meta-analysis involving 26 randomized trials demonstrated that an additional reduction of 1 mmol/L in LDL-C levels was associated with a 22% reduction in the incidence of major vascular events, a 10% reduction in all-cause mortality, and a 20% reduction in CHD mortality [21]. Accordingly, current guidelines emphasize the importance of intensifying lipid-lowering treatment and achieving very low LDL-C levels in patients at high risk of cardiovascular events, including those with recent ACS or MVD or those undergoing coronary revascularization [8, 22]. For lipid management in patients with a recent ACS, most guidelines favor a step-by-step regimen that includes early administration of statins at a high-intensity dose, followed by combination with ezetimibe. PCSK9 inhibitors will be taken into account if the recommended treatment targets have not been achieved [8, 22]. With this scheme, PCSK9 inhibitor therapy was not considered for ACS patients with substantially elevated LDL-C levels until several months after the index event. Considering statin intolerance [11], the delayed effect of statins, as well as inertia with regard to dose maximization [13], ACS patients frequently fail to attain guideline-recommended LDL-C levels despite intensive statin treatment [9, 10]. Nevertheless, the risk of recurrent cardiovascular events is greatest in the early post-ACS period [23]. This highlights the potential necessity for a fast-acting and more potent drug, in addition to statins, to rapidly and significantly lower LDL-C levels and further improve cardiovascular outcomes.

The FOURIER (Further Cardiovascular Outcomes Research with PCSK9 Inhibition in Subjects with Elevated Risk) trial [15] demonstrated that the evolocumab combined with intensive statin treatment, as compared with statins alone, significantly decreased the risk of major ischemic cardiovascular events among patients with stable ASCVD. Similarly, a meta-analysis of 39 randomized controlled trials including 66,478 patients indicated that PCSK9 inhibitors lowered the risk of MI by 20% (95% CI, 14–26%; *p* < 0.0001), ischemic stroke by 22% (95% CI, 11–33%; *p* = 0.0005) and coronary revascularization by 17% (95% CI, 11–22%; *p* < 0.0001) [24]. In these studies, however, evolocumab treatment was only considered in subjects with off-target LDL-C levels after receiving maximally tolerated statin therapy. Given the high risk of early recurrent ischemic events after ACS, we put forward a novel scheme of the early addition of PCSK9 antibody treatment to statins in patients who were not expected to achieve guideline-recommended LDL-C targets with high-intensity statins alone. The results indicated that evolocumab plus statin therapy lowered LDL-C levels to a mean of 0.75 mmol/L and reduced the risk of recurrent cardiovascular events.

EPIC-STEMI (Effects of Acute, Rapid Lowering of Low Density Lipoprotein Cholesterol with Alirocumab in Patients with ST Segment Elevation Myocardial Infarction Undergoing Primary PCI) [25] is a recently conducted randomized, double-blind, and sham-controlled clinical trial with the aim of investigating the impact of PCSK9 inhibitors added to high-intensity statin therapy on LDL-C levels in STEMI patients who underwent PCI. At a median of 45 days, the PCSK9 inhibitor alirocumab reduced LDL-C levels by 72.9% compared to 48.1% in the sham control group. More patients achieved the European dyslipidemia guideline target of LDL-C ≤1.4 mmol/L in the alirocumab group (92.1%) than the sham control group (56.7%) [25]. In our study, the one-month follow-up showed a decrease in LDL-C levels of 77.02% in the evolocumab group, which is consistent with the findings of the EPIC-STEMI study. However, the standard-of-care group only showed a decrease of 26.20%, which is lower than that observed in the EPIC-STEMI study. We speculate that the differences in inclusion criteria between these two studies may have caused this discrepancy. The EPIC-STEMI study enrolled patients who received PCSK9 inhibitors regardless of their baseline LDL-C levels. In contrast, our study focused on early administration of PCSK9 inhibitors in patients who remained suboptimal after receiving statins for at least one week or presented with very high LDL-C levels upon admission. It is plausible to assume that the LDL-C lowering effect in our standard-of-care group was moderate, with lower rates of achieving LDL-C goals. However, regardless of the inclusion criteria, both studies demonstrated a substantial reduction in LDL-C levels with the use of PCSK9 inhibitors. Our research has shown that long-term treatment with PCSK9 inhibitors can significantly improve the prognosis of patients with ACS and with MVD undergoing PCI.

Multiple clinical trials have shown that patients with MVD experience a significantly increased risk of recurrent cardiovascular events. A register-based study conducted in patients with MI demonstrated that CHD severity was a critical risk factor for the composite endpoint of MI, stroke, or cardiovascular mortality within 1 year (3-vessel disease: odds ratio and 95% CI, 4.18, 3.66–4.77; 2-vessel disease, 3.23, 2.81–3.72) [4]. In a cohort study involving 37,674 patients undergoing coronary angiography for CHD, patients with multivessel obstructive CHD had a substantially higher 1-year risk of MI than those without apparent CHD (3-vessel obstructive CHD: HR and 95% CI, 19.5, 9.9–38.2; 2-vessel obstructive CHD, 16.5, 8.1–33.7) [5]. In the PROSPECT (Providing Regional Observations to Study Predictors of Events in the Coronary Tree) trial [6], 20.4% of patients with ACS who underwent PCI and current evidence-based treatments had recurrent major adverse cardiovascular events within 3 years, which were equally divided between those associated with culprit lesions and those associated with non-culprit lesions. Accordingly, ACS patients with MVD undergoing PCI represent a very high-risk group. A secondary analysis from the FOURIER trial showed that patients with stable ASCVD and with MVD are at substantially higher risk for major cardiovascular events despite maximally tolerated statin therapy, and derive significant risk reduction with LDL-C-lowering treatment with evolocumab [7]. In the present study, relative to statins alone, the addition of evolocumab to statin treatment substantially lowered the risk of primary and principal secondary efficacy end points, mainly due to a reduction in the rate of MI. The incidence of MI in both target and non-target vessels tended to be reduced in the evolocumab group compared with the control group, suggesting potential benefits of evolocumab in plaque stabilization and inhibition of neo-atherosclerosis in both culprit and non-culprit lesions. These results corresponded well with the findings in the HUYGENS (High-Resolution Assessment of Coronary Plaques in a Global Evolocumab Randomized Study) trial [26], which showed that evolocumab along with statin treatment resulted in favorable effects on stabilization and regression of coronary atherosclerosis compared with statins alone, as evidenced by a significant increase in minimum fibrous cap thickness and reduction in maximum lipid arc and macrophage index. Based on these findings, it would be reasonable to preferentially target evolocumab treatment for ACS patients with MAD undergoing PCI.

Our subgroup analysis showed that the effect of evolocumab on the primary outcome was greater in individuals with low hs-CRP levels than those with high hs-CRP levels, which is consistent with a previous study that demonstrated the impact of inflammation on the vascular benefits of PCSK9 inhibitors [27]. This prospective observational study aimed to investigate the influence of neutrophil-to-lymphocyte ratio (NLR) on the cardiovascular benefit of PCSK9 inhibitors in familial hypercholesterolemia (FH) subjects with ASCVD. The study found that only FH subjects with low NLR experienced a significant reduction in pulse wave velocity (PWV) after six months of PCSK9 inhibitors therapy, while no significant changes were observed in the high-NLR group [27]. A previous study has demonstrated a positive association between NLR and hs-CRP levels in individuals with high risk of cardiovascular disease [28]. The authors noted in their discussion that despite intensive lipid-lowering therapy, the interplay between neutrophils and lymphocytes promoted a significant systemic inflammatory state [27, 29]; furthermore, a recent study has found that NLR can serve as a valuable prognostic biomarker independently predicting all-cause death and major adverse cardiovascular events [27, 28]. The findings of these studies could elucidate why only subjects with lower inflammatory states were able to benefit from PCSK9 inhibitors therapy, while those with higher inflammatory states did not show significant improvements in prognosis. Further randomized controlled trials will be necessary to substantiate our preliminary discoveries.

It is worth noting that recent studies have revealed that the protective effects of evolocumab may not be solely attributed to the reduction of LDL-C levels, but also potentially arise from pleiotropic effects. Nicola Ferri *et al*. [30] have identified five supporting evidences supporting the investigation of PCSK9 inhibitors as a rapid and aggressive treatment option for patients with ACS. Firstly, during ACS, levels of circulating PCSK9 increase. Secondly, higher levels of circulating PCSK9 have been directly correlated with platelet reactivity, a crucial factor in the recurrence of ischemic cardiovascular events [31]. Thirdly, PCSK9 is correlated with activation of macrophage, inflammation, and endothelial dysfunction within plaques [32]. Fourth, PCSK9 concentration is related to inflammation during the acute phase of ACS [32, 33]. Finally, statin therapy can rapidly and sometimes markedly increase PCSK9 levels [34]. Therefore, it can be speculated that the cardiovascular protective effects of evolocumab may not only arise from reducing LDL-C levels, but also from its capacity to inhibit platelet activation, alleviate plaque inflammation and macrophage activation, improve endothelial function, and attenuate the increased PCSK9 levels induced by statin therapy. Additional clinical and fundamental research investigations must be formulated to validate our hypotheses.

The current randomized controlled trials (RCTs) evaluating evolocumab in the field of cardiovascular disease have mainly focused on patients with high ischemic risk, such as those with a history of MI, MVD, ACS, or non-ST-segment elevation myocardial infarction [15, 18, 26]. These studies indicated that evolocumab has significant potential to lower LDL-C levels, stabilize and reverse plaque vulnerability, and ultimately decrease the incidence of cardiovascular events in these high-risk populations [15, 18, 26]. However, our trial experienced a permanent discontinuation rate of 8.0% (25 patients) due to the high cost of evolocumab treatment. Fortunately, the price of evolocumab in China has significantly dropped to $43 per 140 mg, thus easing the financial burden for patients with ASCVD. Considering the reasonable price and superior therapeutic efficacy of evolocumab, physicians are increasingly considering the administration of evolocumab in low- or moderate-risk patients, including those with single-vessel disease, stable CHD, intermediate coronary stenosis, and type A lesions. In view of the benefits of evolocumab in reducing the risk of recurrent cardiovascular events, this shift in LDL-C-lowering has the potential to bring enormous clinical benefits to Chinese patients with ASCVD. It will also provide a wealth of real-world evidence for the efficacy, safety and feasibility of evolocumab in ASCVD patients with low, moderate or high-risk status.

Some limitations should be acknowledged. Due to the retrospective nature of this study, the use of evolocumab was at the discretion of the attending physician, which may introduce a potential selection bias. Although the multivariable Cox regression model minimized the potential confounders related to the study endpoints, residual unmeasured confounders cannot be eliminated. Consequently, future multicenter, prospective, randomized trials are required to determine the optimal intensive lipid-lowering strategy, especially in patients at very high ischemic risk.

## 5. Conclusions

Among ACS patients with MVD taken for PCI, evolocumab initiated in-hospital along with statin treatment lowered LDL-C levels to a mean of 0.75 mmol/L and reduced the risk of recurrent cardiovascular events, with favorable safety and tolerability.

## Data Availability

The datasets used and/or analyzed during the current study are available from the corresponding author on reasonable request.
